# Egalitarianism and altruism in health: some evidence of their relationship

**DOI:** 10.1186/1475-9276-13-13

**Published:** 2014-02-06

**Authors:** Ignacio Abásolo, Aki Tsuchiya

**Affiliations:** 1 Departamento de Economía de las Instituciones, Estadística Económica y Econometría, Facultad de Ciencias Económicas y Empresariales, Universidad de La Laguna, Campus de Guajara, Tenerife, Spain; 2 Department of Economics and School of Health and Related Research, University of Sheffield, 30 Regent Street, Sheffield S1 4DA, UK

**Keywords:** Egalitarianism, Altruism, Socioeconomic health inequalities, Blood donation

## Abstract

**Background:**

Egalitarianism and altruism are two ways in which people may have attitudes that go beyond the narrowly defined selfish preferences. The theoretical constructs of egalitarianism and altruism are different from each other, yet there may be connections between the two. This paper explores the empirical relationship between egalitarianism and altruism, in the context of health.

**Methods:**

We define altruism as individual behaviour that aims to benefit another individual in need; and egalitarianism as a characteristic of a social welfare function, or a meta-level preference. Furthermore, we specify a model that explains the propensity of an individual to be egalitarian in terms of altruism and other background characteristics. Individuals who prefer a hypothetical policy that reduces socioeconomic inequalities in health outcomes over another that does not are regarded ‘egalitarian’ in the health domain. On the other hand, ‘altruism’ in the health context is captured by whether or not the same respondents are (or have been) regular blood donors, provided they are medically able to donate. Probit models are specified to estimate the relationship between egalitarianism and altruism, thus defined. A representative sample of the Spanish population was interviewed for the purpose (n = 417 valid cases).

**Results:**

Overall, 75% of respondents are found to be egalitarians, whilst 35% are found to be altruists. We find that, once controlled for background characteristics, there is a statistically significant empirical relationship between egalitarianism and altruism in the health context. On average, the probability of an altruist individual supporting egalitarianism is 10% higher than for a non-altruist person. Regarding the other control variables, those living in high per capita income regions have a lower propensity and those who are politically left wing have a higher propensity to be an egalitarian. We do not find evidence of a relationship between egalitarianism and age, socioeconomic status or religious practices.

**Conclusion:**

Altruist individuals have a higher probability to be egalitarians than would be expected from their observed background characteristics.

## Background

Egalitarianism and altruism are both attitudes that go beyond immediate selfish concerns. Yet, as we describe below, altruism is about the nature of one’s own utility function, while egalitarianism is about the kind of Social Welfare Function (SWF) one has a meta-level preference for. Thus, at a theoretical level, there is no reason to assume that the two are associated with each other. However, the two concepts may be supported by the same people in the real world. One reason why this may happen is because both concepts are associated with social norms and practices. There are established social norms that prescribe individuals should be helpful to others and that equality should be promoted: indifference is regarded as disgraceful and inequality as reprehensible. Furthermore, it might be expected that altruistic people, who are willing to sacrifice own resources (money, time, blood) to benefit others in need, are also more likely to believe in egalitarian redistribution from those who have more to those who have less. But if so, are altruists actually more likely to be egalitarian, beyond what would be expected given their socioeconomic characteristics? This is what we want to test empirically. There are a number of studies that looked at the presence of these in experimental settings [1,2]. The objective of this paper is to examine empirically how these two are related in the health context and using survey data.

Health is a context where egalitarianism and altruism may play an important role in individuals’ preferences. For example, the supply of blood in many places is based on voluntary donations with no financial reward, and thus on altruism. On the other hand, publicly funded health systems in many places hold as an important policy goal the reduction of socioeconomic health inequalities, and thus are egalitarian. At the same time, it has been shown that for example, in Spain, while the majority of the general public support egalitarianism [3], only a much lower proportion of the population donate blood regularly [4].

What has not been analysed so far is the potential connection between egalitarianism and altruism in the health context which will contribute towards understanding the true motivations of individuals’ preferences. In particular, to the best of our knowledge, studies have not explicitly considered and measured the effect of altruism on the preference for egalitarian policies in the health domain.

In terms of determinants of egalitarian preferences in health other than altruism, sex and political affiliation have been explored by [5], finding that amongst Swedish politicians, women and, as expected, those left wing have relatively more sympathies towards equity. In addition, [6] find that age, per capita income of region of residence and the way the question is administered to respondents have an effect on the propensity to choose an egalitarian policy; while sex and socioeconomic status (proxied by education level and household income) have no significant effect.

This paper explores empirically the relationship between egalitarianism and altruism, in the context of health. The second section presents the concepts of altruism and egalitarianism. The third section then presents the methods of the empirical study. The fourth section reports the results and the final section concludes.

### Concepts of altruism and egalitarianism

#### Altruism

Let us begin first with altruism, because this concerns the nature of individual preferences, and thus relates to a more fundamental level for economic theory than egalitarianism, which relates to an interpersonal and less fundamental level. Standard microeconomic theory begins by assuming that *homo economicus* is ‘selfish’ and ‘rational’. This does not carry any judgmental implications (i.e. it does not imply that the economic agent is morally suspect), but simply means that economics aims to model individual choice by assuming that individuals will make decisions aiming to maximise their own individual utility subject to their personal budget constraints.

However, real humans are capable of unselfish behaviour. To accommodate this, economists can take a closer look at the individual utility function, and introduce the concept of ‘caring externalities’ [7,8]. This is when the utility of an individual (*i*) is a function of (amongst other things such as *i*’s own consumption) the welfare of another individual (*j*) where *j* is likely to be somebody regarded by *i* to be ‘in need’. There is a related literature on ‘interdependent utilities’, which is where the externalities are reciprocal [9,10]; but this also includes the case of envy (negative externality) and not just altruism (positive externality). Once a utility function with caring externalities is built, economists can model and predict the decisions of an individual with such a utility function (for instance, see Jones-Lee [11]). In a way, the individual with caring externalities remains selfish and rational in the sense that they maximise their own utility. We may call this instrumental altruism: behaviour that benefits others, but is fundamentally motivated by selfishness (see for example McGuire et al. [12] or Mooney [13]).

If society is made up of just two individuals *i* and *j*, then this framework works well to explain altruistic behaviour. However, when society is made up of three or more individuals, the concept of public goods becomes relevant, and alongside this, the possibility of free-riding [7,14,15]. If *i*’s utility is affected by *j*’s welfare, then *i*’s utility can be improved if some third party (say, *k*), also with caring externalities, took action to improve *j*’s welfare (unless the only way *i*’s utility improved from *j*’s welfare was when the improvement was due to *i*’s own action; see next paragraph). This means that, because *k* cannot stop *i* benefiting from *j*’s welfare improved by *k*, and vice versa, *j*’s welfare is now a public good. If this became common knowledge, then both *i* and *k* may not act to improve *j*’s welfare. Each may count on the other to act and try to free-ride, thus leading to an under-supply of the public good. The implication of this is that if individuals are fully rational and information is complete, then altruistic behaviour in a world with more than three individuals becomes increasingly difficult to explain, even with caring externalities.

There are other approaches that aspire to go beyond instrumental altruism, and assume that human beings are capable of going above oneself and of behaving in line with another’s welfare completely disregarding selfish interests because it is intrinsically ‘the right thing to do’. The debate at this point becomes somewhat semantic. If the individual gains any satisfaction, or ‘warm glow’ [16], from feeling one is doing the right thing (or, if the individual is to suffer regret for not doing the right thing), then it becomes difficult to distinguish this from instrumental altruism. However, it may be noted that unlike its instrumental version, the altruism here is not affected by the public good scenario above, because for the positive externality to arise for *i*, it is not enough to simply know *j*’s welfare has been improved; the improvement has to be attributed to *i*. Then, improving *j*’s welfare becomes a means to a more fundamental end, to do good, and in this respect, again, altruism can be regarded as being something instrumental rather than intrinsic (but at a different level).

For practical purposes, it makes sense to define altruism as behaviour that aims to benefit another individual in need. It seems unnecessary to require altruism to be incompatible with self-interest or to demand that altruism involves bringing net loss to oneself. Just like the technical term ‘selfish’ does not carry any moral connotation, the technical term ‘instrumental altruism’ need not carry any implication that it is morally less worthy than an act of intrinsic value. Thus, we regard altruism as something that goes beyond immediate selfish concerns, but not necessarily something that goes against immediate selfish concerns, or something that is incompatible with wider selfish concerns.

In this paper we will consider blood donation as a proxy for altruism in the health context. Donating blood for transfusion to total strangers has featured in the literature as a classic example of altruistic behaviour [17-21]. However, blood donation is a peculiar case because of its own nature. There is limited supply of blood, and since every unit of blood transfused to a particular patient is a unit of blood that cannot be used for another patient, there is opportunity cost associated with its use. It is a highly perishable good with a strict ‘use by’ date beyond which it should not be put to therapeutic use, so effective management of its stocks is important. In addition, it is a resource that can only be procured by drawing it from another human being; modern biotechnology has not yet achieved the synthesis of artificial blood. While blood donation entails some time costs, very mild pain, no health benefits, and possibly some self-satisfaction to the donor, it can have substantial health benefits to the recipient. One complication associated with analysing blood donation is that not everybody is medically eligible to donate. However, accounting for this is arguably less problematic than devising an appropriate way to adjust for variation in budget constraints when analysing monetary donations.

#### Egalitarianism

Egalitarianism implies equality of something (i.e. the ‘equalisand’), and thus involves comparing across at least two parties. The key issue in any debate of egalitarianism concerns the question, equality of what [22], and may concern capabilities, income, utility, achieved health, and so on. The objective of this paper is not to argue for a specific equalisand: we will start by simply taking ‘equality of outcomes’ as the equalisand for now, and focus on health later.

Even then, this may lead to some confusion, if egalitarianism is defined with respect to resulting distributions of outcomes alone, independently of the mechanism behind it. For instance, a distribution-neutral social welfare function (SWF) can lead to egalitarian outcomes if individuals share the same risk averse utility function. This is because such a utility function has diminishing marginal utility, and social welfare will be maximised if the marginal good is distributed to the person with the largest marginal utility, which is the least well off person. Over time, this set up will result in everybody achieving an equal distribution of the equalisand, and thus an unintended egalitarian outcome.

A somewhat less powerful but similar example is a distribution-neutral SWF combined with individuals with caring externalities towards those with low welfare. Then again, under certain conditions, social welfare maximisation and inequality reduction will coincide, and over time an egalitarian distribution will be achieved, without anybody being egalitarian. However, trying to base egalitarianism on individual-level preferences such as caring-based positive externalities seems contrived, because it is not clear why the type of externality should be restricted to those that are caring. A group of individuals with envy-based negative externalities could also achieve an egalitarian distribution in the long run without intending to.

Such an apparent paradox where a distribution-neutral social welfare function leads to egalitarian distributions can be avoided if the concept of egalitarianism is reserved for the aggregate level. Then, egalitarianism will be about the functional form of the SWF, and not about the functional form of individual utility functions (e.g. diminishing marginal utility) or what is included in these (e.g. caring externalities). Then, there are two paths to take. One is to say because the above distribution-neutral SWF gives equal weight to everybody’s welfare, it is itself egalitarian, and thus there is no paradox in the first place. However, taking this route is not compatible with egalitarianism defined as equality of outcomes. The other is to define egalitarianism as explicit efficiency-equality trade-offs, and to require preferences with diminishing marginal rate of social substitution (MRSS), which is in effect the approach used in conventional welfare economics [9,23]. This is the definition used in this paper.

The next issue then is the mechanism for determining the MRSS. One approach would be to base it on revealed (collective) preference. If data are available where analysts can compare numerous actual policy decisions made in the real world, then a SWF may be fitted to the data and an estimate of MRSS obtained. If successful, this would allow the identification of the SWF in the descriptive sense. Another approach is to base it on stated or expressed preferences of individuals using hypothetical states of the world, involving different distributions of outcomes, and asking the individuals to indicate their choices. Individuals faced with such an exercise may approach it in two ways.

One would be to form a view regarding which position one may find oneself in, and to choose from this ‘private or personal perspective’ in line with what would be to one’s own benefit (with or without caring externalities as may be the case). This is what happens under the Rawlsian setup of the veil of ignorance with extreme risk aversion [24]. However, this does not involve any reference to a SWF. In other words, the maximin rule is not derived from the so-called Rawlsian SWF. It is the other way round, and it is the Rawlsian SWF that is a product of maximin, which in turn is the rational choice of extremely risk averse individuals behind the veil of ignorance. Thus in our terminology, the egalitarianism of the maximin rule is unintended. If individuals are completely risk neutral, then the veil of ignorance will lead to a distribution-neutral (and therefore non-egalitarian) SWF.

If we require egalitarianism to be defined by a SWF with diminishing MRSS and we wish to elicit this through stated preference, then we need respondents to engage in the exercise with reference to the ‘societal or citizens perspective’, in line with how, in their judgment, society should allocate resources. In other words, the parameters of an egalitarian SWF cannot be derived by looking at the individual’s own utility function, but can only be captured by some meta-level preference along the lines of Sen’s ‘meta-ranking’ [25], Hare’s ‘critical thinking’ [26], or the ‘social perspective’ [27].

In the context of health, defining egalitarianism in terms of equal outcomes means equalising health outcomes. There can be further variations to this: equalising health across individuals, or across population groups? And if across population groups, which groups? Or, what is the measure of health used? Is it inequality in health at any point in time, or in lifetime health? For the purpose of this paper, we will use reducing inequality in life expectancy at birth across socio-economic groups as the working example of egalitarianism in health.

## Methods and data

### Methods

We specify a model that explains the propensity for an individual to be egalitarian in the above sense. An underlying (or latent) variable (E^*^) represents an individual’s propensity to choose, or not choose, an *egalitarian* health policy that reduces inequality in outcomes as opposed to a policy that does not. We examine the association between this propensity for egalitarianism and *altruism* (*A*), controlling for a series of observable background characteristics. As a proxy for altruism, we consider whether the individual is or has ever been a regular blood donor (provided they are/were medically capable of doing so). This is not the only way in which altruism could be captured but we believe that this should be a reasonable proxy, particularly in the health context.

In line with previous evidence outlined above, we hypothesise that people’s attitudes towards egalitarianism will be explained by their demographic, socioeconomic, ideological, together with religious characteristics. Particularly, starting with demographic factors, sex (*Sex*) and age (*Age*) are considered. Secondly, since we are dealing with attitudes regarding socio-economic health inequalities, we may expect there to be some pattern by the respondent’s socio-economic status: proxies used^a^ to explore this possibility are education (*Edu*), whether the individual is unemployed (*Unempl*). Thirdly, the per capita income of the region of residence (*Reginc*) is used to control for regional variation by grouping the region by mean income. Fourthly, we consider that people’s attitude towards egalitarianism can be affected by both political affiliation or ideology (*Ideol*) and religious practice (*Relig*).

Thus, the model can be written as:

(1) $$E_{i}^{*}=E\left(A_{i},\mathrm{Se}x_{i},\mathrm{Ag}e_{i},\mathrm{Ed}u_{i},\mathrm{Unemp}l_{i},\mathrm{Regin}c_{i},\mathrm{Ideo}l_{i},\mathrm{Reli}g_{i}\right)+\epsilon_{i}$$

In model [Eq. 1], the *i* subscripts represent individual respondents, and *ϵ*_*i*_ captures unobserved influences, which are assumed to have a standard normal distribution with zero mean and constant variance.

In practice, E^*^is unobserved. Instead, we observe *E*_*i*_, which is a dummy variable representing whether or not the individual actually chooses the egalitarian policy. Therefore, it is the realization of a binomial process defined by:

$$E_{i}=1\;\mathrm{if}\;\left[E^{*}>0\right]$$

So, if the individual’s propensity to be egalitarian is positive (E^*^> 0) s/he will choose the egalitarian policy (*E*_*i*_ = 1), and if otherwise (E^*^≤ 0) s/he will not (*E*_*i*_ = 0).

The estimation process will be undertaken through probit regressions. Likelihood ratio (LR) tests and Reset specification tests will be carried out to appraise the appropriateness of the different functional forms.

We have information to distinguish individuals who are (or have been) regular blood donors from those who are not (or have not been). Furthermore, we can identify those individuals who are not blood donors due to medical or health reasons. It would be inappropriate to classify this latter group as non-altruists, since we have incomplete information regarding whether they would have been blood donors if their medical/health restriction did not exist. Therefore we treat them as missing and exclude them from the analysis. If such medical restrictions on blood donation applied at random, then amongst this sub-population the proportion of those who would otherwise have donated blood would be the same as the proportion of those who donate blood amongst the rest of the population; and the effect of *altruism* and the rest of covariates on *egalitarianism* would not be significantly different. Therefore, excluding them from the analysis would not introduce a bias.

However, if this is not at random, then regression analyses that exclude these respondents will be biased (i.e. selection bias would occur). Tests for selection bias and correction, if necessary, are undertaken estimating a probit with sample selection [28]. The probit with sample selection works in a manner very similar to the Heckman model [29] except that the response variable is binary. For this selection model, let us assume an underlying (unobserved) variable that determines the selection of individuals into groups, i.e. *P*_*i*_ = 1 when > threshold, and *P*_*i*_ = 0 when ≤ threshold, represents the probability of the individual to be able to donate blood. It is assumed that is a linear function of some of the exogenous variables in model [Eq. 1], in addition to some identifying variables:

(2) $$D_{i}^{*}=D\left(\mathrm{Se}x_{i},\mathrm{Ag}e_{i},\mathrm{Ed}u_{i},\mathrm{Reli}g_{i},\mathrm{Healt}h_{i},\mathrm{Rura}l_{i},\mathrm{Prin}s_{i}\right)+u_{i}$$

The identifying variables include the health state of the individual (*Health*_*i*_), whether the individual is resident in a rural area (*Rural*_*i*_), and whether the individual has private insurance (in addition to public health insurance) (*Prins*_*i*_). The main criteria used here for proposing this set of identifying variables is that the variables have an impact on the probability to be able to donate blood but are unrelated to the individual’s preference for egalitarian policies. *u*_*i*_ is a random error term normally distributed with zero mean and constant variance. Selection bias occurs when there is correlation between *D* and *ϵ* (and therefore between *ϵ* and *u*); in other words, when unobservable factors that influence the eligibility to be a blood donor are also influencing the probability to choose the egalitarian option. If so, selection bias will be corrected. To check whether selection bias is absent we will test, firstly, whether *ρ* (the correlation of residuals) is significantly different from zero: if the covariance between *ϵ* and *u* is significantly different from zero, then we cannot reject that there is no selection bias. In addition, a comparison of the estimates of the main egalitarian model with and without the blood donor selection model is undertaken: a large change in the coefficients, a change of the sign of the coefficients, or a change in the statistical significance of the coefficients between the models with and without selection will indicate the existence of selection bias.

Finally, our model [Eq. 1] is built to analyse the effect of *altruism* on *egalitarian* attitudes of individuals, and relies on the assumption of exogeneity of the right-hand-side variables (such as political affiliation, religious practice and others, in addition to being altruist). Given that altruism is the main focus of this paper, we will consider the potential endogeneity of this covariate only. in particular, although it is assumed that altruism may affect egalitarianism, it could be the case that egalitarian attitudes also have an effect on altruism. If this is the case, we would have an endogeneity problem caused by simultaneity, where conventional estimators will be biased and inconsistent. Exogeneity of the covariate *altruist* is tested through the Smith and Blundell test [30]. This involves a two-stage procedure where *altruist* is first modelled using instruments and the residuals from this regression are entered in the second regression modelling *egalitarian*. The test examines whether these residuals are significant in the second regression. If not, then the null hypothesis that *altruist* is exogenous cannot be rejected, and the model specified as [Eq. 1] is accepted. However, if the test result is significant, this means the null is rejected so that there is an endogeneity problem. As identifying binary variables we consider whether the individual is an organ donor (*Organ*_*i*_) and whether the individual abstained in the previous general elections (*Abstenc*_*i*_). To be valid, firstly, the instruments should be correlated with *altruism* (individually and jointly significance tests are undertaken): we anticipate that those who are organ donors are more likely to donate blood as well; and regarding abstention in the previous elections, we expect those individuals who did not vote are less concerned about collective actions and therefore are less likely to donate blood. And secondly, the instruments should be uncorrelated with the error term of the egalitarian equation: this is tested with the Amemiya-Lee-Newey test of overidentifying restrictions. Once the validity of the instruments is established, if the Smith and Blundell test statistic is not significant then it would suggest that the estimations are consistent and unbiased. Otherwise, simultaneity should be addressed by constructing instrumental variables for this endogenous regressor.

### Data and variables definition

The questions were designed by the authors and the data were collected during 2004 by ASEP/JDS (a commercial survey company) in Spain, a country with a National Health Care System characterised by universal coverage and tax funding. A survey of 801 individuals over 18 years of age was undertaken. Face to face interviews were assigned across the 17 “Comunidades Autónomas” (“Regions” for short), reflecting the local resident population proportionally. Within each of the Regions, interviews were randomly allocated so that the achieved sample will be representative of the general Spanish population in terms of socio-demographic characteristics. In general, 49% of the individuals were male, with average age of 45 (SD 17.9); and 51% female, with average age of 48 (SD 18.6).

**Figure 1:**
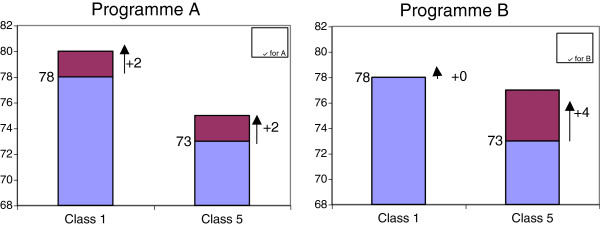
The visual aid given to respondents.

Regarding egalitarianism, the interview questionnaire included one question in which the respondent is asked to think as if s/he was a decision maker who has to choose between two alternative health programmes. Figure 1 reproduces the visual aid used in the interviews. Initially, the respondent is presented with a 5-year difference in life expectancy at birth between higher and lower socioeconomic classes (78 and 73 years respectively). Social class is defined on the basis of occupation: high social class is represented by professions like doctors or lawyers, whilst low social class is represented by road sweepers or cleaners. Programme A would increase the life expectancy of both classes by 2 years each (and therefore maintain the current 5-year gap in life expectancy); whereas programme B would increase the life expectancy of the worse-off class by 4 years (and reduce the current inequality). The respondents are informed that both programmes have exactly the same cost. Then the respondent is asked as follows: *As you can see, programme A targets both social classes equally and programme B targets the lower social class. Please, tick the corresponding box indicating whether you would choose programme A, programme B, or if you consider that both programmes are equally good.* The dependent variable *egalitarian* takes the value 1 if the individual prefers the programme that reduces health inequality, and 0 if the individual does not.

With respect to the approach to altruism, the respondent is asked whether s/he is, or has been, a regular blood donor. Those who reply “no” are asked for the main reason from a short list. Those who select “because of medical reasons” at this stage are excluded from the analysis as explained above. The binary independent variable *altruist* takes the value 1 if individual *i* is or has been a regular blood donor, 0 if otherwise. Note that in Spain, blood donations have always been voluntary with no monetary (or in-kind) remunerations. Regarding the rest of factors that we have controlled for, age has been categorised in four dummy variables: *age (18–35)* (baseline category), *age (36–45)*, *age (46–55)*, *age (56–65)*, and *age (66*+*).* The binary variable *female* indicates whether the individual is female or not. Regarding the socioeconomic variables, education is recorded by level of schooling and has been categorised in three dummy variables *primary education* those with primary school education or less (baseline category), *secondary education*, and *university education*; and the dummy variable *unemployment* indicates whether the individual is currently working. Per capita income in the region of residence is captured by three dummies: *high income region* (Madrid, Navarra or País Vasco), *low income region* (Andalucía or Extremadura) and *middle income region* (the rest of Spain, the omitted category). Political affiliation is recorded by the binary variable *left wing* (those who report themselves being centre-left, left or extreme left wing). Finally, the binary variable *no religion* indicates that the respondent does not practice a religion.

Regarding the identifying variables for the probit with sample selection, the self-reported measures of health include a categorical indicator that records whether individual considered their general health during the twelve months prior to the survey to be very poor, poor, fair, good and very good. So we have three dummies *fair health* (for those with very poor, poor or fair health, used as the baseline), *good health* and *very good health*. Population size of the area of residence is proxied by *small area* indicating whether the individual lives in an area of 10,000 or less inhabitants. Last, *private health insurance* records whether the survey respondent has private health insurance (in addition to the public health insurance). Finally, regarding the instruments to test for exogeneity of the covariate *altruist*, we have considered the binary variable *organ donor* indicating whether the respondent reports being registered as an organ donor; and the binary variable *abstained* indicating whether the respondent abstained in the 2004 general elections.

### Research ethics clearance

Research ethics clearance was given by Research Ethics and Animal Welfare Committee at the University of La Laguna, March 2013 (ref. CEIBA2013-0060).

## Results

Descriptive statistics are reported in Table 1. Out of the 801 respondents involved in the relevant questions, item non-response leads to 235 missing cases, which corresponds to 29% of the entire data, leaving 566 valid observations. As can be seen, the distribution of background characteristics across the whole sample and the sample used in the analysis are very similar Of these, 149 individuals report that they cannot donate blood because of medical reasons, and are excluded from the analysis. Regarding the remaining 417 individuals, 75% are egalitarians (i.e. prefer the egalitarian policy) and 35% are altruists (i.e. report to be or have been regular blood donors). If we categorise the individuals in the sample according to these two variables of interest (see Table 2), most of the respondents are egalitarian and non-altruists (47%), followed by those who are egalitarian and altruists (28%), those who are neither egalitarian, nor altruists (18%) and finally those who are non-egalitarians but altruists (7%). The corresponding chi-squared test shows that the egalitarian and altruist characteristics are not independent, and there is a statistically significant relationship between both categorical variables (*p* < 0.05).

**Table 1:** Summary statistics

	**Whole sample**		**Valid cases**	**Sample used in the probit**
			**N = 566**	**N = 417**
**Variable**	**N**	**Mean**	**Mean**	**Mean**
Egalitarian	770	.735	.751	.751
Altruist	800	.244	.258	.350
Female	801	.506	.510	.470
Age (18–35)*	801	.327	.327	.381
Age (36–45)	801	.195	.207	.225
Age (46–55)	801	.132	.133	.120
Age (56–65)	801	.149	.150	.132
Age (66+)	801	.197	.184	.141
Primary education*	799	.343	.304	.259
Secondary education	799	.538	.565	.604
University education	799	.119	.131	.137
Unemployed	799	.064	.067	.065
Middle income region*	801	.605	.643	.643
High income region	801	.192	.170	.175
Low income region	801	.202	.187	.182
Left wing	654	.564	.564	.580
No religión	764	.450	.472	.513
Fair health*	801	.262	.223	.161
Good health	801	.634	.668	.722
Very good health	801	.102	.109	.118
Small area	801	.242	.219	.230
Private health insurance	796	.139	.164	.149
Organ donor	791	.096	.101	.101
Abstention	717	.162	.085	.089

*Denotes baseline category.

**Table 2:** Cross frequencies egalitarians/altruists (n = 417)

	**Altruist**	**Non-altruist**
Egalitarian	118 (28.3%)	195 (46.8%)
Non-egalitarian	28 (6.7%)	76 (18.2%)

The four cells add up to 100%.

Pearson chi2 (1) = 3.984; p = 0.046.

Now, the question is whether this empirical relationship holds when we model egalitarianism as a function of altruism and other background characteristics. Probit estimations for the egalitarian model [Eq. 1] are shown in the first column of Table 3. The reset test shows that there is no evidence of mis-specification: the *chi*-squared test statistic is 0.180 with a *p*-value above conventional levels (*p* = 0.672). Overall, the model is statistically significant (*p* < 0.05) but the McFadden R-squared statistic is just 0.075; however, as is often the case when the probit is applied to cross-sectional data (with modest sample size) the goodness of fit is low. Estimates indicate that altruism has a significant and positive effect on the propensity to support egalitarianism (*p* < 0.05), once controlling for other factors.

**Table 3:** Probit results for egalitarian

	**Coeff.**	**Rob.std.Err.**	**Mg.effect**
Altruist	.331 (**)	.152	.097
Female	−.038	.139	−.011
Age (36–45)	.161	.185	.047
Age (46–55)	−.076	.231	−.024
Age (56–65)	.251	.240	.071
Age (66+)	.395	.262	.108
Secondary education	−.066	.192	−.020
University education	−.380	.248	−.127
Unemployed	−.268	.284	−.088
High income region	−.518 (**)	.184	−.175
Low income region	−.261	.188	−.084
Left wing	.485 (**)	.147	.151
No religion	.212	.147	.065
Constant	.375	.257	

N = 417.

Wald chi2(13) = 33.84; Prob > chi2 = 0.001; McFadden’s R2 = 0.075.

Reset test:chi2(1) = 0.18; Prob > chi2 = 0.672.

**p-value < 0.05; *p-value < 0.1.

Dependent variable: egalitarian.

The third column of Table 3 shows the probit marginal effects. Given that all of the covariates are binary variables, the marginal effects are interpreted as the percentage point change in the probability of being an egalitarian resulting from a discrete change in the explanatory variable. Particularly, other things equal, the marginal effect of altruism on egalitarianism is 0.097 indicating that on average, the probability of an altruist individual supporting egalitarianism is 10% higher than for a non-altruist person. Regarding the other control variables, those living in high per capita income regions have a lower propensity to be egalitarian by about 17% compared to those living in middle income regions (*p* < 0.05). On the other hand, as expected, those who are politically left wing have a significantly higher probability to be an egalitarian compared to those who are centre-right. In particular, the probability of a left wing individual being egalitarian is on average 15% higher than the reference individual, other things being equal (*p* < 0.05). Finally, gender, age, socioeconomic status and religious practices do not have a significant association with the probability to be egalitarian.

Estimates for the egalitarian model with sample selection [Eq. 2] to accommodate medical restrictions on blood donation can be seen in Table 4. The correlation coefficient (*ρ*) is not statistically different from zero (*p* = 0.285), suggesting that we cannot reject the null hypothesis that there is no selection bias. In addition, the sign, the magnitude and the *t*-ratios of coefficients of this egalitarian model with selection are quite close to those of the initial egalitarian model without selection [Eq. 1], with the only exception of the *t*-ratio of the binary variable aged 66 or more. Regarding the potential endogeneity of altruism, the Smith-Blundell test of exogeneity indicates that we cannot reject the null hypothesis that *altruist* is exogenous: the test statistic is 0.00548 and the *p*-value is 0.941. The instruments *organ_don* and *abstenc* appear to be valid: *F*-test show that the instruments are jointly significant (chi2 (2) = 19.11 (*p* < 0.05) and *t*-tests also indicate that they are individually significant in the *altruist* equation (*p* < 0.05); in addition, the Amemiya-Lee-Newey chi-squared test is 2.070 with a *p*-value of 0.1503, showing that the instruments are uncorrelated with the error term of the egalitarian model (i.e. therefore, providing further support for instrument validity).

**Table 4:** Results on probit with sample selection

**Egalitarian (E)**			**Being able to donate blood (D)**		
	**Coeff.**	**R.std.Err**		**Coeff.**	**R.std.Err.**
Altruist	.328 (**)	.147	Female	−.346 (**)	.124
Female	.031	.145	Age (36–45)	−.232	.186
Age (36–45)	.194	.186	Age (46–55)	−.654 (**)	.207
Age (46–55)	−.061	.251	Age (56–65)	−.576 (**)	.209
Age (56–65)	.356	.239	Age (66+)	−.663 (**)	.217
Age (66+)	.564 (**)	.280	Secondary education	.097	.156
Secondary education	−.061	.192	University education	.081	.225
University education	−.363	.247	No religion	.113	.130
Unemployed	−.267	.267	Good health	.646 (**)	.145
High income region	−.497 (**)	.175	Very good health	.412 (*)	.236
Low income region	−.238	.185	Small area	.244	.157
Left wing	.455 (**)	.151	Private health insurance	−.374 (**)	.163
No religion	.191	.156	Constant	.648 (**)	.259
Constant	.456 (*)	.266	/Athrho	−.563	.552
			Rho	−.510	.408

N = 566; censored = 149.

Wald chi2(13) = 34.10; Prob > chi2 = 0.001.

LR-test indep. eqns: rho = 0; chi2(1) = 1.14; Prob > chi2 = 0.2853.

**p-value < 0.05; *p-value < 0.1.

## Discussion

Egalitarianism and altruism are both attitudes that go beyond immediate selfish concerns. However, the two theoretical constructs are different from each other, and yet, there may be connections between the two at the empirical level.

Would we expect that those who are altruist to be also egalitarian? In this study we have found that in the context of health just under half the respondents are egalitarian (i.e. have a preference for an egalitarian policy) but not altruist (i.e. report not to be or have been a regular blood donor); over a quarter are both egalitarian and altruists; and a fifth are neither egalitarian nor altruist. This relationship is confirmed through a model of egalitarianism: the probability of an altruist individual being an egalitarian as well is significantly higher than for a non-altruist person, controlling for other background characteristics.

Regarding the other covariates, gender was found to have no significant impact on the propensity to be egalitarian, in line with the results of [6] but unlike [5] which found that women are relatively more sympathic towards equity. In line with the findings of [6], the socioeconomic status was also found to have no effect on attitudes towards socioeconomic inequalities in health, which might be indicating a non-selfish response behaviour, so the questionnaire might be picking up correctly the societal preferences, as aimed. Different conclusions are reached by Hudson and Jones [31] who showed that those more educated are less averse to higher taxes for health policy issues. Also in line with [6], living in a high per capita region is negatively associated with a preference for egalitarian policies. However, unlike it, neither age nor religious practice had any significant effect. Finally, as expected, those politically left wing are more likely to prefer egalitarian policies, evidence that was also found previously by [5] and [6].

We acknowledge that blood donation is only one of several possible altruistic behaviours, and is not a perfect measure. However, it is a behaviour that has been used in the literature as an example of anonymous altruistic giving in the context of health, [20,21,32,33]. There may be individuals who are altruists but do not donate blood because of medical reasons; we have excluded these cases from the analysis and undertaken analysis to check for potential sample selection. The analysis did not distinguish between the remaining reasons for not donating, which included: aversion to needles; not having thought about it; because others already do it; and any other reason. We decided simply to consider whether or not the individual is or has been a regular blood donor as a proxy for altruism, taken at face value. The implication is that those who are willing to donate but for whom it is highly inconvenient to do so (e.g. because there are no facilities nearby) are treated as not altruistic, which may be problematic. However, the proportion of respondents selecting “other” reason for not donating was under 1% of the whole sample.

The overall sample size diminishes at each stage of the analysis, which may lead to concerns over, for example, geographical representativeness of the analysis sample. Due to small sample size in some of the regions, controlling by region was not practical. Instead, regions were grouped by per capita income. Test of independence (or equality of proportions) indicated that the null hypothesis that the proportions are the same in the whole sample and the analysis sample cannot be rejected (chi2(2) = 2.065; *p* = 0.356). Furthermore, the sample selection model indicates that incomplete information on those who are not blood donors because of medical reasons does not introduce a significant bias. In addition, the exogeneity test rejects the possibility of biased and inconsistent estimators due to simultaneity between the altruist and egalitarian variables.

The data we have are based on an interview survey, and we only have what the respondent has told the interviewer. Egalitarianism is measured by responses to a question based on a hypothetical choice between two policy scenarios in health, and one may criticise its substance. However, it should be noted that we define egalitarianism as a meta-preference. This means that there is no real-world opportunity where true, or revealed, preferences can be revealed through observable behaviours. Altruism on the other hand is measured by response to a question on respondent behaviour, regarding blood donation. This may be more valid than the question on egalitarianism, but there may be issues of interpretation and/or recall. For example, we have not given the exact definition of ‘regular’. If respondents were biased by social norms and were trying to appear pleasant to the interviewer, then this would affect the two key variables in the same way: social norms will inflate both egalitarian preferences and altruistic blood donation. Furthermore, the fact that the egalitarian question preceded the blood donation question in the interview may have influenced the responses to the latter.

Compared to experimental settings, which have been predominant in the monetary payoffs contexts, where relevant scenarios can be manipulated to explore the relevant parameters, interviews are very crude. However, the objective of this paper is to probe about their real world behaviour and to ask for their reasons for it, which is better suited to interview surveys.

Overall, egalitarianism and altruism in health measured in these ways are associated with each other. Those who are or have been regular blood donors are more likely to choose the egalitarian policy than would be expected from their observed background characteristics.

### Endnote

^a^Household income was also available but given the high rate of missing cases of this variable (40%) and the resulting final sample size, it is not used in the analysis.

## Competing interests

The authors declared no potential conflicts of interest with respect to the research, authorship, and/or publication of this article.

## Authors’ contributions

Both IA and AT have contributed to the conception and design of the research and to the analysis and interpretation of the data. Both have also been involved in drafting and revising the manuscript, have given final approval to the version to be published and have agreed to be accountable for all aspects of the research related to accuracy and integrity of any part of the work. Both authors read and approved the final manuscript.
